# Social vulnerability and genetic service utilization among unaffected BRIDGE trial patients with inherited cancer susceptibility

**DOI:** 10.1186/s12885-025-13495-4

**Published:** 2025-01-31

**Authors:** Jemar R. Bather, Melody S. Goodman, Adrian Harris, Guilherme Del Fiol, Rachel Hess, David W. Wetter, Daniel Chavez-Yenter, Lingzi Zhong, Lauren Kaiser-Jackson, Rachelle Chambers, Richard Bradshaw, Wendy Kohlmann, Sarah Colonna, Whitney Espinel, Rachel Monahan, Saundra S. Buys, Ophira Ginsburg, Kensaku Kawamoto, Kimberly A. Kaphingst, on behalf of the BRIDGE research team

**Affiliations:** 1 Center for Anti-Racism, Social Justice & Public Health, New York University School of Global Public Health, 708 Broadway, 9th Floor, New York, NY 10003 USA; 2 Department of Biostatistics, New York University School of Global Public Health, New York, NY USA; 3 Department of Biomedical Informatics, Spencer Fox Eccles School of Medicine, University of Utah, Salt Lake City, UT USA; 4 Department of Population Health Sciences, Spencer Fox Eccles School of Medicine, University of Utah, Salt Lake City, UT USA; 5 Department of Internal Medicine, Spencer Fox Eccles School of Medicine, University of Utah, Salt Lake City, UT USA; 6 Center for Health Outcomes and Population Equity (HOPE), Huntsman Cancer Institute, Salt Lake City, UT USA; 7 Division of Hematology-Oncology, Perelman School of Medicine at the University of Pennsylvania, Philadelphia, PA USA; 8 Department of Medical Ethics and Health Policy, Perelman School of Medicine at the University of Pennsylvania, Philadelphia, PA USA; 9 Department of Communication, University of Minnesota Duluth, Duluth, MN USA; 10 Huntsman Cancer Institute, Salt Lake City, UT USA; 11 Perlmutter Cancer Center, NYU Langone Health, New York, NY USA; 12 Clinical Cancer Genetics Service, VA Medical Center National TeleOncology, Durham, NC USA; 13 Breast/Gynecologic System of Excellence, VA Medical Center National TeleOncology, Durham, NC USA; 14 Division of Medical Oncology, Huntsman Cancer Institute, Salt Lake City, UT USA; 15 Division of Oncology, Huntsman Cancer Institute, Salt Lake City, UT USA; 16 Center for Global Health, National Cancer Institute, Rockville, MD USA; 17 Department of Communication, University of Utah, Salt Lake City, UT USA

**Keywords:** Health technology, Cancer predisposition syndromes, Decision making, Cancer prevention, Early detection, User interaction, Patient experience, Population screening, Carrier screening, Cancer disparities

## Abstract

**Background:**

Research on social determinants of genetic testing uptake is limited, particularly among unaffected patients with inherited cancer susceptibility.

**Methods:**

We conducted a secondary analysis of the Broadening the Reach, Impact, and Delivery of Genetic Services (BRIDGE) trial at University of Utah Health and NYU Langone Health, involving 2,760 unaffected patients meeting genetic testing criteria for inherited cancer susceptibility and who were initially randomized to either an automated chatbot or an enhanced standard of care (SOC) genetic services delivery model. We used encounters from the electronic health record (EHR) to measure the uptake of genetic counseling and testing, including dichotomous measures of (1) whether participants initiated pre-test cancer genetic services, (2) completed pre-test cancer genetic services, (3) had genetic testing ordered, and (4) completed genetic testing. We merged zip codes from the EHR to construct census tract-weighted social measures of the Social Vulnerability Index. Multilevel models estimated associations between social vulnerability and genetic services utilization. We tested whether intervention condition (i.e., chatbot vs. SOC) moderated the association of social vulnerability with genetic service utilization. Covariates included study arm, study site, age, sex, race/ethnicity, language preference, rural residence, having a recorded primary care provider, and number of algorithm criteria met.

**Results:**

Patients living in areas of medium socioeconomic status (SES) vulnerability had lower odds of initiating pre-test genetic services (adjusted OR [aOR] = 0.81, 95% CI: 0.67, 0.98) compared to patients living in low SES vulnerability areas. Patients in medium household vulnerability areas had a lower likelihood of completing pre-test genetic services (aOR = 0.80, 95% CI: 0.66–0.97) and having genetic testing ordered (aOR = 0.79, 95% CI: 0.63–0.99) relative to patients in low household vulnerability areas. We did not find that social vulnerability associations varied by intervention condition.

**Conclusions:**

These results underscore the importance of investigating social and structural mechanisms as potential pathways to increasing genetic testing uptake among patients with increased inherited risk of cancer. Census information is publicly available but seldom used to assess social determinants of genetic testing uptake among unaffected populations. Existing and future cohort studies can incorporate census data to derive analytic insights for clinical scientists.

**Trial registration:**

BRIDGE was registered as NCT03985852 on June 6, 2019 at clinicaltrials.gov.

**Supplementary Information:**

The online version contains supplementary material available at 10.1186/s12885-025-13495-4.

## Background

Recent advances in artificial intelligence have led to increased implementation of automated, patient-directed conversational agents (i.e., chatbots) to increase genetic service utilization [1–16]. There is a growing literature on the potential utility of chatbots compared with conventional methods (i.e., in-person genetic counseling appointments) in the cancer/clinical genetics setting [13, 14]. Chatbots have been used to provide pre-test genetics education, assess genetic cancer risk, offer genetic counseling, and disseminate genetic testing results to family members [1–5, 8–15]. Despite studies of chatbot engagement in various patient populations [2, 3, 5, 11, 13–15], research on social determinants of genetic testing uptake is limited, particularly among unaffected patients with inherited cancer susceptibility [17–19].

Results from the Early Detection of Genetic Risk (EDGE) study showed that individual-level socioeconomic factors, particularly lower education and household income levels, were associated with decreased interest in hereditary cancer genetic testing among individuals with high hereditary cancer risk [17]. Yet, more studies are needed to further clarify determinants of genetic testing uptake at a structural level (e.g., social vulnerability) [18–23]. It is well-established that these structural patterns are associated with increased cancer mortality and later stage cancer diagnosis for historically minoritized populations [24]. Investigating the interrelationship between social factors and patient-directed conversational agents would provide an added window into understanding roles that chatbots could play in increasing genetic testing uptake among unaffected communities with inherited cancer risk [18]. Also, such research could help understand the potential impact of chatbots on equity in use of genetic testing [18]. Identifying social and structural barriers can also inform novel multilevel interventions coordinated by genetic counselors, physicians, and health communication specialists [25].

Motivated by these research gaps, we constructed a multilevel design using American Community Survey estimates and secondary data from the Broadening the Reach, Impact, and Delivery of Genetic Services (BRIDGE) trial, which compared two genetic service delivery models (chatbot vs. enhanced standard of care [SOC]) [1, 16]. In the present study, we examined the association of area-level social vulnerability with uptake of genetic counseling and testing among unaffected patients at risk for inherited cancer susceptibility. We tested whether intervention condition (i.e., chatbot vs. SOC) moderated the association of social vulnerability with genetic service utilization. We hypothesized that individuals living in areas with higher social vulnerability would have lower uptake of genetic services and that this association would be attenuated among chatbot arm participants. This attenuation may occur because chatbots can mitigate some barriers associated with social vulnerability, such as eliminating the need for time off work or transportation to appointments, thus potentially increasing access to genetic services.

## Methods

### Study design and setting

This observational study is based on a secondary analysis of data from the BRIDGE study, a patient-level randomized (1:1) equivalence trial comparing genetic service uptake across two genetic service delivery models (automated, patient-directed conversational agent [chatbot] versus enhanced SOC) [1, 16]. Details of the full study protocol have been previously reported [1, 16, 26]. Briefly, the Genetic Cancer Risk Detector (GARDE) software platform identified unaffected primary care patients eligible for evaluation for hereditary cancer syndromes at two large healthcare systems (University of Utah Health [UHealth] and NYU Langone Health [NYULH]) [27–29]. GARDE is an open-source population health platform that scans patients’ electronic health records (EHRs) for cancer family history information to determine their eligibility for cancer genetic testing [27–29]. The eligibility criteria were based on the 2018 National Comprehensive Cancer Network (NCCN) guidelines for genetic evaluation of hereditary ovarian, pancreas, breast, colorectal, and/or prostate cancers [27–30]. BRIDGE trial inclusion criteria required individuals to (1) meet the NCCN genetic evaluation criteria, (2) be 25 to 60 years old, (3) speak English or Spanish, (4) have received primary care in the UHealth or NYULH systems within the past three years (2017–2019), (5) not have any cancer history except non-melanoma skin cancer, (6) not have engaged in any hereditary cancer-related genetic counseling or testing services, and (7) have or be willing to create a MyChart patient portal account in Epic® [1]. We defined primary care as internal or family medicine at both sites, as well as primary care visits in obstetrics and gynecology at UHealth. The UHealth and NYULH Institutional Review Boards (IRBs) approved the BRIDGE trial study protocol as a single IRB protocol (IRB 00115509). BRIDGE was registered as NCT03985852 on June 6, 2019 at clinicaltrials.gov. This secondary analysis adhered to the Declaration of Helsinki and the Strengthening the Reporting of Observational Studies in Epidemiology guidelines [31].

### Analytic sample

**Fig. 1:**
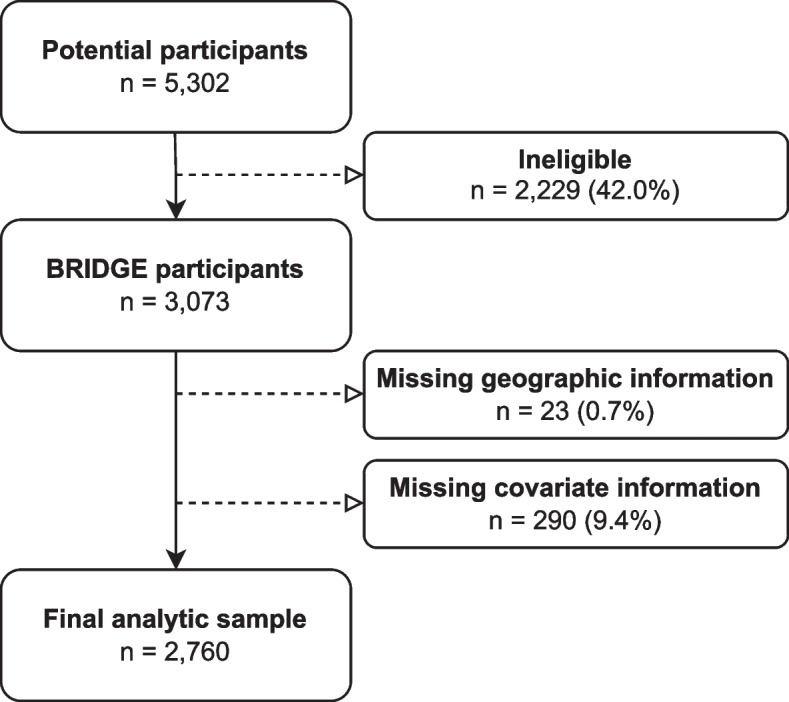
Derivation of the analytic sample, Broadening the Reach, Impact, and Delivery of Genetic Services (BRIDGE) randomized controlled trial, 2020–2023

Figure 1 describes the analytic sample derivation. Between 2020 and 2023, a total of 5,302 potential participants were randomly selected to participate in the BRIDGE trial [1]. Of these, 42% were ineligible. Of the 3,073 eligible study participants, we excluded 10% due to missing data on having a recorded primary care provider (*n* = 1), age (*n* = 2), sex (*n* = 7), race/ethnicity (*n* = 280), or geographic information (*n* = 23). The final analytic sample comprised 2,760 unaffected patients at increased risk for inherited cancer.

### Study arms

#### Automated, patient-directed conversational agent

A patient portal message recommending genetic services was sent to patients in the chatbot intervention arm with a hyperlink to launch the chatbot providing pre-test genetics education. BRIDGE trial coordinators, genetic counselors, and health communication specialists developed the pre-test genetics education script, which was delivered using the Invitae chatbot platform [1, 4, 32]. The chatbot’s script was based on the process of a SOC pre-test genetic counseling appointment. Chatbot arm patients who did not respond to the initial patient portal message received a one-week follow-up reminder, with up to two additional follow-up reminders by genetic counseling assistants via telephone. Patients who requested genetic testing at the end of the chatbot pre-test education were contacted by genetic counseling assistants to discuss the patient’s decision to pursue genetic testing and to confirm the patient’s family history. If the patient confirmed their intent to move forward with genetic testing, the genetic counseling assistant placed a request in the laboratory portal for the patient to be sent a saliva collection kit to their home or arranged for the patient to come to an in-person facility for a blood draw. Pan-cancer, multigene panel tests for 34–36 genes were conducted by Clinical Laboratory Improvement Act-certified and New York State approved (NYULH patients) commercial laboratories.

#### Enhanced standard of care

Patients in the enhanced SOC arm also received a patient portal message recommending genetic services. Instead of receiving the genetics education chatbot hyperlink, these patients were prompted to call and schedule a pre-test genetics counseling appointment at their study site or via phone call. One-week follow-up reminders were sent with up to two additional telephone call reminders by genetic counseling assistants offering to schedule an appointment. Certified genetic counselors provided clinical standard of care for pre-test genetic counseling services to SOC arm patients. These appointments occurred predominately by phone, but could also be in person based on patient preference. As in the chatbot arm, if the patient decided to move forward with genetic testing, a genetic counseling assistant placed an order with a genetic testing laboratory. Those having genetic counseling by phone had a saliva kit sent to their home, and those seen in person had the option to give a sample of blood or saliva at the visit.

### Uptake of genetic counseling and testing

We used encounters from the EHR of all study participants to measure the uptake of genetic counseling and testing. Outcomes of interest included dichotomous measures of (1) whether participants initiated pre-test cancer genetic services (defined as clicking the hyperlink for pre-test genetics education to launch the chatbot in the chatbot arm, or scheduling a pre-test counseling appointment with a genetic counselor at their study site in the SOC arm), (2) completed pre-test cancer genetic services (defined as chatbot arm patients completing the chatbot pre-test genetics education or SOC arm patients completing the pre-test genetic counseling appointment), (3) had genetic testing ordered, and (4) completed genetic testing.

### Social vulnerability

We obtained patients’ zip codes from the EHR to construct census tract-weighted social vulnerability measures according to the Social Vulnerability Index developed by the Centers for Disease Control and Prevention [33]. We analyzed the socioeconomic status (SES) and household vulnerability metrics which are derived from 2018–2022 American Community Survey 5-year estimates. SES vulnerability is based on the following census estimates: percent of population living below 150% of the federal poverty level, percent of population that is unemployed, percent of population experiencing housing cost burden, and percent of population without a high school diploma [33]. Household vulnerability is based on the following census estimates: percent of households with adults aged 65 years or older, percent of households with children aged 17 years or younger, percent of households that have a civilian with a disability, percent of households that are single-parent households, and percent of households with limited English language proficiency [33]. We proportionally weighted each metric using census tract weights from the US Department of Housing and Urban Development ZIP Code Crosswalk files [34]. Index scores range from 0 to 1, indicating an area’s social vulnerability ranking (e.g., 0.50 represents the 50th percentile). Higher scores indicated greater social vulnerability. We categorized these continuous measures into low, medium, and high groups.

### Covariates

We selected covariates based on prior literature from our group as well as others [35–42], which informed the selection of individual-level variables important to control for in order to examine the independent effect of social vulnerability. Covariates extracted from the EHR included age (measured continuously), sex (female, male), race/ethnicity (non-Hispanic White, non-Hispanic Black, Hispanic, non-Hispanic Other), language preference (English, Spanish), and whether the patient had a recorded primary care provider. We assessed urbanicity (urban versus rural) by merging zip codes from the EHR with the 2010 Rural–Urban Commuting Area Codes established by the US Department of Agriculture [43]. We dichotomized the number of GARDE algorithm criteria for genetic testing that were met (only one versus multiple) [27–30].

### Statistical analysis

We computed descriptive statistics for all variables using the gtsummary R package [44]. We reported counts and percentages for categorical measures. Means and standard deviations described continuous measures. We performed bivariate analyses for all variables by SES and household vulnerability using Pearson’s Chi-squared, Wilcoxon rank sum, and Fisher’s exact tests [45]. Multilevel logistic regression models were estimated using generalized estimating equations [46]. We employed an exchangeable working correlation structure to account for clustering within zip codes. Covariate-adjusted associations were obtained for each social vulnerability metric with the uptake of genetic counseling and testing outcomes. We included interaction terms to test whether social vulnerability associations differed by study arm. Odds ratios (ORs) and 95% confidence intervals (CIs) were tabulated. We also computed marginal effects for interpretability [47]. Statistical significance was assessed as a two-sided alpha of 0.05. We used R Version 4.4.0 to perform all statistical analyses [48].

## Results

### Sample characteristics

Table 1 summarizes descriptive characteristics among the overall sample. Of the 2,760 participants, 1,361 were from NYULH and 1,399 were from UHealth. The average age of the study participants was 44 years (SD = 10). Most of the sample were female (73%), non-Hispanic White (75%), preferred to speak English (99%), had a recorded primary care provider recorded in the EHR (76%), and lived in an urban area (96%). Ninety-four percent met only one algorithm criterion. Regarding the outcomes of interest, 30% initiated pre-test genetic services, 25% completed pre-test genetic services, 17% had genetic testing ordered, and 13% completed genetic testing.

**Table 1:** Sample characteristics, overall and by socioeconomic status vulnerability, Broadening the Reach, Impact, and Delivery of Genetic Services (BRIDGE) trial, 2020–2023

**Characteristic**	**Overall** N = 2,760	**Socioeconomic status vulnerability**			***p*-value**^1^
		**Low percentile** $\le$**32.2** *n* = 1,391	**Medium percentile** **32.3 to 64.4** *n* = 908	**High percentile** **64.5 to 96.7** *n* = 461	
**Study arm, No. (%)**					0.28
* Enhanced standard of care*	1,364 (49%)	687 (49%)	463 (51%)	214 (46%)	
* Chatbot*	1,396 (51%)	704 (51%)	445 (49%)	247 (54%)	
**Study site, No. (%)**					**< 0.001**
* NYU Langone Health*	1,361 (49%)	597 (43%)	438 (48%)	326 (71%)	
* University of Utah Health*	1,399 (51%)	794 (57%)	470 (52%)	135 (29%)	
**Age, Mean (SD)**	44 (10)	44 (10)	44 (10)	43 (10)	0.21
**Female sex, No. (%)**	2,023 (73%)	985 (71%)	683 (75%)	355 (77%)	**0.009**
**Race/ethnicity, No. (%)**					**< 0.001**
* non-Hispanic White*	2,068 (75%)	1,176 (85%)	665 (73%)	227 (49%)	
* non-Hispanic Black*	202 (7%)	39 (3%)	65 (7%)	98 (21%)	
* Hispanic*	313 (11%)	91 (7%)	125 (14%)	97 (21%)	
* non-Hispanic Other*	177 (6%)	85 (6%)	53 (6%)	39 (8%)	
**English language preference, No. (%)**	2,725 (99%)	1,384 (99%)	890 (98%)	451 (98%)	**0.001**
**Algorithm criteria met, No. (%)**					0.46
* Multiple*	176 (6%)	95 (7%)	57 (6%)	24 (5%)	
* Only one*	2,584 (94%)	1,296 (93%)	851 (94%)	437 (95%)	
**Has a recorded primary care provider, No. (%)**	2,104 (76%)	1,050 (75%)	682 (75%)	372 (81%)	**0.047**
**Urban residence, No. (%)**	2,653 (96%)	1,335 (96%)	860 (95%)	458 (99%)	**< 0.001**
**Initiated pre-test services, No. (%)**	838 (30%)	442 (32%)	252 (28%)	144 (31%)	0.11
**Completed pre-test genetic services, No. (%)**	688 (25%)	368 (26%)	208 (23%)	112 (24%)	0.15
**Had genetic testing ordered, No. (%)**	459 (17%)	250 (18%)	141 (16%)	68 (15%)	0.15
**Completed genetic testing, No. (%)**	358 (13%)	191 (14%)	111 (12%)	56 (12%)	0.49

^1^Pearson's Chi-squared test; Kruskal–Wallis rank sum test

### Sample characteristics by socioeconomic status and household vulnerability

We observed statistically significant differences in several demographic characteristics by SES vulnerability (Table 1). Compared to the low and medium groups, the high SES vulnerability group had the greatest proportions of females, non-Hispanic Black patients, those with a recorded primary care provider, individuals who lived in an urban area, and NYULH patients (Table 1). We observed relatively similar patterns by household vulnerability (Table 2).

**Table 2:** Sample characteristics by household vulnerability, Broadening the Reach, Impact, and Delivery of Genetic Services (BRIDGE) trial, 2020–2023

**Characteristic**	**Household vulnerability**			***p*-value**^1^
	**Low percentile** $\le$**31.6** *n* = 1,243	**Medium percentile** **31.7 to 63.1** *n* = 1,266	**High percentile** **63.2 to 94.8** *n* = 251	
**Study arm, No. (%)**				0.36
* Enhanced standard of care*	631 (51%)	607 (48%)	126 (50%)	
* Chatbot*	612 (49%)	659 (52%)	125 (50%)	
**Study site, No. (%)**				**< 0.001**
* NYU Langone Health*	607 (49%)	582 (46%)	172 (69%)	
* University of Utah Health*	636 (51%)	684 (54%)	79 (31%)	
**Age, Mean (SD)**	43 (10)	44 (10)	44 (10)	0.09
**Female sex, No. (%)**	874 (70%)	950 (75%)	199 (79%)	**0.002**
**Race/ethnicity, No. (%)**				**< 0.001**
* non-Hispanic White*	1,011 (81%)	938 (74%)	119 (47%)	
* non-Hispanic Black*	47 (4%)	103 (8%)	52 (21%)	
* Hispanic*	108 (9%)	142 (11%)	63 (25%)	
* non-Hispanic Other*	77 (6%)	83 (7%)	17 (7%)	
**English language preference, No. (%)**	1,233 (99%)	1,246 (98%)	246 (98%)	0.10
**Algorithm criteria met, No. (%)**				**0.009**
* Multiple*	98 (8%)	62 (5%)	16 (6%)	
* Only one*	1,145 (92%)	1,204 (95%)	235 (94%)	
**Has a recorded primary care provider, No. (%)**	912 (73%)	992 (78%)	200 (80%)	**0.005**
**Urban residence, No. (%)**	1,195 (96%)	1,215 (96%)	243 (97%)	0.82
**Initiated pre-test services, No. (%)**	390 (31%)	370 (29%)	78 (31%)	0.49
**Completed pre-test genetic services, No. (%)**	332 (27%)	297 (23%)	59 (24%)	0.15
**Had genetic testing ordered, No. (%)**	224 (18%)	197 (16%)	38 (15%)	0.20
**Completed genetic testing, No. (%)**	173 (14%)	155 (12%)	30 (12%)	0.40

^1^Pearson's Chi-squared test; Kruskal–Wallis rank sum test; Fisher's exact test

### Social vulnerability, pre-test genetic services, and completion of genetic testing

Table 3 presents the adjusted associations between SES vulnerability and genetic services utilization. We found that patients living in areas of medium SES vulnerability had lower odds of initiating pre-test genetic services (adjusted OR [aOR] = 0.81, 95% CI: 0.67, 0.98) compared to patients living in low SES vulnerability areas. This translated to a marginal effect of −0.04 (95% CI: −0.08, −0.01; Table 4).

**Table 3:** Adjusted associations between socioeconomic status vulnerability and genetic service utilization

	**Initiated pre-test genetic services**		**Completed pre-test** **genetic services**		**Had genetic** **testing ordered**		**Completed** **genetic testing**	
	OR	95% CI	OR	95% CI	OR	95% CI	OR	95% CI
**Socioeconomic status vulnerability**								
* Low (ref.)*								
* Medium*	0.81	0.67, 0.98	0.82	0.67, 1.02	0.84	0.66, 1.06	0.87	0.68, 1.12
* High*	0.94	0.74, 1.21	0.90	0.68, 1.18	0.79	0.57, 1.09	0.86	0.61, 1.21
**Study arm**								
* Enhanced standard of care (ref.)*								
* Chatbot*	1.09	0.93, 1.28	1.11	0.94, 1.33	0.81	0.67, 1.00	0.93	0.74, 1.16

Models adjusted for study site, age, sex, race/ethnicity, language preference, residence, has a recorded primary care provider, and algorithm criteria met

*OR* Odds ratio, *CI* Confidence interval

**Table 4:** Estimated conditional marginal effects of socioeconomic status vulnerability on genetic service utilization

	**Initiated pre-test genetic services**		**Completed pre-test** **genetic services**		**Had genetic** **testing ordered**		**Completed** **genetic testing**	
	Marginal effect	95% CI	Marginal effect	95% CI	Marginal effect	95% CI	Marginal effect	95% CI
**Socioeconomic status vulnerability**								
* Low (ref.)*								
* Medium*	−0.04	−0.08, −0.01	−0.04	−0.07, 0.00	−0.02	−0.06, 0.01	−0.02	−0.04, 0.01
* High*	−0.01	−0.07, 0.04	−0.02	−0.07, 0.03	−0.03	−0.07, 0.01	−0.02	−0.05, 0.02

*CI* Confidence interval

We observed that patients in medium household vulnerability areas had a lower likelihood of completing pre-test genetic services (aOR = 0.80, 95% CI: 0.66–0.97) and having genetic testing ordered (aOR = 0.79, 95% CI: 0.63–0.99) relative to patients in low household vulnerability areas (Table 5). The marginal effect in the “completed pre-test genetic services” model was −0.04 (95% CI: −0.08, −0.01; Table 6), and the marginal effect in the “had genetic testing ordered” model was −0.03 (95% CI: −0.06, −0.01; Table 6). The interaction terms between study arm and social vulnerability metrics were not statistically significant and were not included in the final models. All final models controlled for study arm, study site, age, sex, race/ethnicity, language preference, residence, having a recorded primary care provider, and number of algorithm criteria met.

**Table 5:** Adjusted associations between household vulnerability and genetic service utilization

	**Initiated pre-test genetic services**		**Completed pre-test** **genetic services**		**Had genetic** **testing ordered**		**Completed** **genetic testing**	
	OR	95% CI	OR	95% CI	OR	95% CI	OR	95% CI
**Household vulnerability**								
* Low (ref.)*								
* Medium*	0.85	0.71, 1.01	0.80	0.66, 0.97	0.79	0.63, 0.99	0.82	0.65, 1.03
* High*	0.92	0.68, 1.26	0.81	0.57, 1.13	0.78	0.53, 1.16	0.80	0.52, 1.23
**Study arm**								
* Enhanced standard of care (ref.)*								
* Chatbot*	1.10	0.93, 1.29	1.12	0.94, 1.34	0.82	0.67, 1.00	0.93	0.74, 1.16

Models adjusted for study site, age, sex, race/ethnicity, language preference, residence, has a recorded primary care provider, and algorithm criteria met

*OR* Odds ratio, *CI* Confidence interval

**Table 6:** Estimated conditional marginal effects of household vulnerability on genetic service utilization

	**Initiated pre-test genetic services**		**Completed pre-test** **genetic services**		**Had genetic** **testing ordered**		**Completed** **genetic testing**	
	Marginal effect	95% CI	Marginal effect	95% CI	Marginal effect	95% CI	Marginal effect	95% CI
**Household vulnerability**								
* Low (ref.)*								
* Medium*	−0.03	−0.07, 0.00	−0.04	−0.08, −0.01	−0.03	−0.06, −0.01	−0.02	−0.05, 0.00
* High*	−0.02	−0.08, 0.05	−0.04	−0.10, 0.02	−0.03	−0.08, 0.02	−0.02	−0.07, 0.02

*CI* Confidence interval

## Discussion

Using data on unaffected patients with inherited cancer risk from two large U.S. healthcare systems, we investigated the associations between social vulnerability and genetic services utilization. We found that patients living in areas of medium SES vulnerability had lower odds of initiating pre-test genetic services than those living in low SES areas. We also observed that patients in medium household vulnerability areas had a lower likelihood of completing pre-test genetic services and having genetic testing ordered compared to patients in low household vulnerability areas. We did not find that the social vulnerability associations differed by study arm.

The significant association between SES vulnerability and initiating pre-test genetic services is consistent with recent findings from a population with increased cancer risk [17]. Using data from the EDGE study [49], Dusic and colleagues found that lower educational attainment and household income predicted lower interest in hereditary cancer genetic testing among primary care patients with high cancer risk [17]. These authors reported that individuals who perceived themselves as having lower social status relative to others in society would be interested in genetic testing if it were free or discounted [17]. The results from the EDGE study and the present study underscore the importance of investigating social and structural mechanisms as potential pathways to increase genetic testing uptake among patients with cancer risk. They also highlight the need for more multilevel studies incorporating individual- and structural-level measures among populations with inherited cancer susceptibility [18, 19].

We additionally found that medium levels of household vulnerability were associated with lower odds of genetic services utilization. The CDC defines household vulnerability as areas with a greater proportion of households composed of senior citizens, adolescents, individuals with disabilities, single parents, and low English language proficiency [33]. As such, genetic testing may be seen as a lower priority and a financial burden for individuals living in areas with medium household vulnerability [17, 50]. Although these findings need to be replicated in national and international healthcare systems, they warrant the consideration of area-level household characteristics in population health algorithms and targeted interventions aimed at increasing genetic testing uptake among communities with increased cancer risk.

Multivariable results suggested that intervention condition (i.e., chatbot vs. SOC) did not significantly impact the association of social vulnerability with genetic service utilization. One possible explanation for these nonsignificant findings is that we did not have sufficient power in this secondary data analysis to detect interaction effects [51]. This warrants future studies with adequate sample sizes to investigate whether automated, patient-directed conversational agents modify the effects of social and structural factors on genetic service utilization. This is critical because chatbots allow individuals to engage with genetic services at any time without requiring scheduled appointments or time off work. Yet, other barriers exists, such as low digital inclusion (e.g., low patient portal use, lack of access to smartphones/computers with internet access, and low digital literacy/digital health literacy) [52]. A key direction for future research is to evaluate whether a text-based approach where patients are sent a text message with a link to a chatbot service may mitigate some of these barriers. Lastly, the use of chatbots for genetic services uptake may mitigate the hesitancies of racial/ethnic and/or gender-minoritized patients to pursue genetic testing through a provider of a different race/ethnicity or gender [53–55].

### Strengths and limitations

Our study possessed several strengths. We merged census data with data on unaffected patients with inherited cancer susceptibility from two large healthcare systems. We examined social determinants of genetic testing uptake, moving beyond the identification of racial/ethnic disparities [18–20, 56, 57]. We applied a semi-parametric modeling approach that accounts for clustering within zip codes and makes fewer assumptions about the data than parametric techniques (e.g., mixed-effects models) [46].

The present study is not without limitations. Our findings may not be generalizable to other healthcare systems with patient population demographics that differ from those of UHealth and NYULH. This secondary data analysis may be subject to unmeasured confounding, measurement error, and collider and selection bias. The GARDE algorithm selected unaffected patients with inherited cancer susceptibility using collected cancer family history information available in the EHR [27–29]. Our recent report identified differences in the availability of family cancer history by race/ethnicity, sex, and language preference, which could have led to bias in the selection of study participants [58]. Future investigations are needed to elucidate the impact of missing cancer family history on selection into randomized trials of genetic testing uptake interventions and the subsequent statistical analyses. Also, the data did not permit determining specific reasons for deciding to proceed or not proceed with testing. Lastly, the observed relationships should not be interpreted as causal.

## Conclusions

In summary, our results contribute to understanding how social structures impact the uptake of genetic services. We analyzed data from the BRIDGE trial to explore the relationship between social vulnerability and genetic testing uptake among unaffected individuals at risk for an inherited cancer susceptibility. We also investigated whether an automated, patient-directed conversational agent modified this relationship. Our findings suggest that future interventions to improve pre-test genetic service initiation should focus on SES vulnerabilities, and interventions to enhance completion of pre-test genetic services and increase genetic testing orders should concentrate on addressing household-level vulnerabilities. Additional analyses are needed to identify whether social vulnerability is a causal pathway for genetic testing uptake among this population. Census information is publicly available but seldom used to assess social determinants of genetic testing uptake among unaffected populations. Existing and future cohort studies can incorporate census data to derive analytic insights for genetic counselors, physicians, and health communication scientists.

## Supplementary Information

Supplementary Material 1.

## Data Availability

The data that support the findings of this study are available from the corresponding author upon reasonable request.

## Abbreviations

EDGE: Early Detection of Genetic Risk
BRIDGE: Broadening the Reach, Impact, and Delivery of Genetic Services
SOC: Standard of Care
GARDE: Genetic Cancer Risk Detector
UHealth: University of Utah Health
NYULH: NYU Langone Health
NCCN: National Comprehensive Cancer Network
HER: Electronic Health Record
IRB: Institutional Review Board
