# Supplementary material for: Social vulnerability and genetic service utilization among unaffected BRIDGE trial patients with inherited cancer susceptibility
Source: BMC Cancer. 2025 Jan 31;25:180. doi: 10.1186/s12885-025-13495-4 (PMC11783932; doi:10.1186/s12885-025-13495-4)
Supplement: Supplementary file 1 — Supplementary Material 1. [file 12885_2025_13495_MOESM1_ESM.docx]

Supplementary materials for *“Social Vulnerability and Genetic Service Utilization Among Unaffected BRIDGE Trial Patients with Inherited Cancer Susceptibility”* by Bather at al.

**Supplemental Table 1.** Adjusted associations between socioeconomic status vulnerability and genetic service utilization, Broadening the Reach, Impact, and Delivery of Genetic Services (BRIDGE) randomized controlled trial, 2020-2023.

|  | **Initiated pre-test genetic services** | | **Completed pre-test  genetic services** | | **Had genetic  testing ordered** | | **Completed  genetic testing** | |
| --- | --- | --- | --- | --- | --- | --- | --- | --- |
|  | OR | 95% CI | OR | 95% CI | OR | 95% CI | OR | 95% CI |
| **Socioeconomic status vulnerability** |  |  |  |  |  |  |  |  |
| *Low (ref.)* |  | | | | | | | |
| *Medium* | 0.81 | 0.67, 0.98 | 0.82 | 0.67, 1.02 | 0.84 | 0.66, 1.06 | 0.87 | 0.68, 1.12 |
| *High* | 0.94 | 0.74, 1.21 | 0.90 | 0.68, 1.18 | 0.79 | 0.57, 1.09 | 0.86 | 0.61, 1.21 |
| **Study arm** |  |  |  |  |  |  |  |  |
| *Enhanced standard of care (ref.)* |  | | | | | | | |
| *Chatbot* | 1.09 | 0.93, 1.28 | 1.11 | 0.94, 1.33 | 0.81 | 0.67, 1.00 | 0.93 | 0.74, 1.16 |
| **Study site** |  |  |  |  |  |  |  |  |
| *NYU Langone Health (ref.)* |  | | | | | | | |
| *University of Utah Health* | 1.44 | 1.20, 1.72 | 1.43 | 1.17, 1.74 | 1.62 | 1.29, 2.03 | 1.39 | 1.09, 1.76 |
| **Age** | 1.00 | 0.99, 1.01 | 1.00 | 0.99, 1.01 | 1.01 | 1.00, 1.02 | 1.01 | 1.00, 1.03 |
| **Sex** |  |  |  |  |  |  |  |  |
| *Female (ref.)* |  | | | | | | | |
| *Male* | 0.96 | 0.79, 1.16 | 1.01 | 0.82, 1.23 | 1.02 | 0.81, 1.28 | 0.96 | 0.74, 1.24 |
| **Race/ethnicity** |  |  |  |  |  |  |  |  |
| *non-Hispanic White (ref.)* |  |  |  |  |  |  |  |  |
| *non-Hispanic Black* | 1.39 | 0.99, 1.94 | 1.21 | 0.84, 1.73 | 1.51 | 1.00, 2.27 | 1.25 | 0.79, 1.98 |
| *Hispanic* | 1.37 | 1.05, 1.79 | 1.38 | 1.04, 1.82 | 1.26 | 0.91, 1.75 | 1.34 | 0.94, 1.92 |
| *non-Hispanic Other* | 1.00 | 0.71, 1.41 | 0.90 | 0.62, 1.31 | 1.00 | 0.65, 1.55 | 1.06 | 0.66, 1.69 |
| **Language preference** |  |  |  |  |  |  |  |  |
| *English (ref.)* |  | | | | | | | |
| *non-English* | 0.90 | 0.42, 1.91 | 0.75 | 0.33, 1.73 | 1.12 | 0.47, 2.71 | 0.70 | 0.24, 2.08 |
| **Residence** |  |  |  |  |  |  |  |  |
| *Rural (ref.)* |  | | | | | | | |
| *Urban* | 1.14 | 0.74, 1.77 | 1.25 | 0.77, 2.03 | 1.60 | 0.88, 2.89 | 1.39 | 0.75, 2.58 |
| **Has a recorded primary care provider** |  |  |  |  |  |  |  |  |
| *No (ref.)* |  | | | | | | | |
| *Yes* | 1.53 | 1.25, 1.89 | 1.54 | 1.23, 1.92 | 1.57 | 1.20, 2.04 | 1.41 | 1.05, 1.88 |
| **Algorithm criteria met** |  |  |  |  |  |  |  |  |
| *Multiple (ref.)* |  | | | | | | | |
| *Only one* | 1.04 | 0.74, 1.46 | 0.93 | 0.66, 1.32 | 1.03 | 0.68, 1.56 | 1.07 | 0.67, 1.70 |
| OR = Odds ratio; CI: Confidence interval | | | | | | | | |

**Supplemental Table 2.** Adjusted associations between household vulnerability and genetic service utilization, Broadening the Reach, Impact, and Delivery of Genetic Services (BRIDGE) randomized controlled trial, 2020-2023.

|  | **Initiated pre-test genetic services** | | **Completed pre-test  genetic services** | | **Had genetic  testing ordered** | | **Completed  genetic testing** | |
| --- | --- | --- | --- | --- | --- | --- | --- | --- |
|  | OR | 95% CI | OR | 95% CI | OR | 95% CI | OR | 95% CI |
| **Household vulnerability** |  |  |  |  |  |  |  |  |
| *Low (ref.)* |  | | | | | | | |
| *Medium* | 0.85 | 0.71, 1.01 | 0.80 | 0.66, 0.97 | 0.79 | 0.63, 0.99 | 0.82 | 0.65, 1.03 |
| *High* | 0.92 | 0.68, 1.26 | 0.81 | 0.57, 1.13 | 0.78 | 0.53, 1.16 | 0.80 | 0.52, 1.23 |
| **Study arm** |  |  |  |  |  |  |  |  |
| *Enhanced standard of care (ref.)* |  | | | | | | | |
| *Chatbot* | 1.10 | 0.93, 1.29 | 1.12 | 0.94, 1.34 | 0.82 | 0.67, 1.00 | 0.93 | 0.74, 1.16 |
| **Study site** |  |  |  |  |  |  |  |  |
| *NYU Langone Health (ref.)* |  | | | | | | | |
| *University of Utah Health* | 1.45 | 1.21, 1.74 | 1.44 | 1.18, 1.76 | 1.66 | 1.32, 2.08 | 1.41 | 1.11, 1.79 |
| **Age** | 1.00 | 0.99, 1.01 | 1.00 | 0.99, 1.01 | 1.01 | 1.00, 1.02 | 1.01 | 1.00, 1.03 |
| **Sex** |  |  |  |  |  |  |  |  |
| *Female (ref.)* |  | | | | | | | |
| *Male* | 0.96 | 0.79, 1.16 | 1.00 | 0.82, 1.23 | 1.01 | 0.80, 1.28 | 0.96 | 0.74, 1.24 |
| **Race/ethnicity** |  |  |  |  |  |  |  |  |
| *non-Hispanic White (ref.)* |  |  |  |  |  |  |  |  |
| *non-Hispanic Black* | 1.40 | 1.01, 1.94 | 1.23 | 0.86, 1.76 | 1.49 | 0.99, 2.23 | 1.25 | 0.80, 1.97 |
| *Hispanic* | 1.35 | 1.04, 1.76 | 1.37 | 1.04, 1.82 | 1.23 | 0.89, 1.71 | 1.33 | 0.94, 1.89 |
| *non-Hispanic Other* | 1.01 | 0.71, 1.42 | 0.90 | 0.62, 1.32 | 1.00 | 0.65, 1.54 | 1.06 | 0.66, 1.69 |
| **Language preference** |  |  |  |  |  |  |  |  |
| *English (ref.)* |  | | | | | | | |
| *non-English* | 0.89 | 0.42, 1.88 | 0.75 | 0.33, 1.71 | 1.11 | 0.46, 2.66 | 0.70 | 0.24, 2.07 |
| **Residence** |  |  |  |  |  |  |  |  |
| *Rural (ref.)* |  | | | | | | | |
| *Urban* | 1.17 | 0.75, 1.81 | 1.26 | 0.77, 2.05 | 1.60 | 0.89, 2.90 | 1.40 | 0.76, 2.60 |
| **Has a recorded primary care provider** |  |  |  |  |  |  |  |  |
| *No (ref.)* |  | | | | | | | |
| *Yes* | 1.55 | 1.26, 1.91 | 1.56 | 1.25, 1.94 | 1.58 | 1.21, 2.06 | 1.43 | 1.07, 1.91 |
| **Algorithm criteria met** |  |  |  |  |  |  |  |  |
| *Multiple (ref.)* |  | | | | | | | |
| *Only one* | 1.05 | 0.75, 1.48 | 0.95 | 0.67, 1.35 | 1.05 | 0.69, 1.59 | 1.09 | 0.69, 1.74 |
| OR = Odds ratio; CI: Confidence interval | | | | | | | | |
